# Diverse biological and engineering strategies towards organ regeneration

**DOI:** 10.1186/s13619-021-00098-0

**Published:** 2021-11-02

**Authors:** Junjie Hou, Qinchao Zhou, Xiaojun Zhu, Jinrong Peng, Jing-Wei Xiong

**Affiliations:** 1 Beijing Key Laboratory of Cardiometabolic Molecular Medicine, Institute of Molecular Medicine, College of Future Technology, Peking University, Beijing, 100871 China; 2 Schools of Biological Sciences and Animal Sciences, Zhejiang University, Hangzhou, 310058 China

## Abstract

Organ regeneration is an important, fascinating, and old topic while much remains unknown in spite of extensive investigations for decades. From March 25th to 27th, 2021, the Third Chinese Symposium on Organ Regeneration took place in the beautiful ocean city of Zhoushan, Zhejiang, China. This biennial conference attracted ~ 300 academic attendees: students, postdoctoral fellows, and principal investigators, in addition to few industrial investigators. The mixed live and virtual talks covered the broad field of organ regeneration from different animal organisms to human organoids, and concluded with some impressive advances on inflammatory signaling, regenerative signaling mechanisms, new technologies, and applications for organ regeneration.

## Main text

Organ regeneration is complex and mysterious, and involves an orchestra of biological processes including inflammation, cell proliferation, transdifferentiation, de-differentiation, and migration. Whisper-like signals are the basic communication methods across diverse cell types. This progressive field cannot continue without efforts from all of its luminaries. Recently, the Third Chinese Symposium on Organ Regeneration on Zhoushan Island, Zhejiang Province, organized by Prof. Jinrong Peng, Prof. Suhong Xu and their colleagues at Zhejiang University, was timely, inspiring and above all, inclusive. The meeting themes covered major areas in the field of animal regeneration, including inflammatory responses, regenerative cell sources and signaling pathways, new technologies, and applications for human diseases. Covering these major topics, each participant aired his or her own views based on a wide range of organs including the liver, heart, limbs, retina, muscle, skin, cartilage, hematopoietic system, and central nervous system. Various model animals were presented in the meeting, from planarians, fish, axolotls, salamanders, mice to monkeys. The meeting also showcased some advanced new technologies. The success of this meeting reminds us of the profound masterworks achieved in bygone days and further lights the path that we will follow.

### Cell sources of regenerative organs

Regenerative capacity varies across species, and even in the same species, different organs possess uneven regenerative ability. Damaged adult organs are capable of regenerating from stem cells (such as hematopoietic tissue, skeletal muscle, skin, and intestine), by transdifferentiation (such as lens), or by de-differentiation and proliferation (such as heart) (Tanaka and Reddien 2011). Regenerative stem cells or progenitors are recognized as an applicable strategy to regenerate upon the injury-induced loss of functional cells, but unfortunately, these stem cells or progenitors do not always exist in all adult organs. Considering this circumstance, the transdifferentiation of pre-existing cells into pluripotent progenitors or their induction into the cell-cycle seems to be an alternative means of accomplishing regeneration. On this topic, Elly Tanaka (Research Institute of Molecular Pathology, Vienna) gave an excellent and comprehensive overview of her decades-long studies on axolotl limb regeneration, noting that the blastemal cell memory and lineage-restricted progenitors are critical for regenerating the limbs (Gerber et al. 2018), and de-differentiation of the limb fibroblasts is the key for a successful regeneration (Lin et al. 2021). Lingjuan He (Westlake University, Hangzhou) reported her recent work on the identification of a group of Ki67-positive proliferative hepatocytes in the midzonal hepatocytes during liver homeostasis and repair (He et al. 2021a). Chengran Xu (Peking University, Beijing) addressed the cell fate of embryonic hepatoblasts by using single-cell RNA-sequencing, noting that hepatoblasts give rise to hepatocytes under control of relevant transcription factors, and the transition into cholangiocytes is easily affected by multiple factors. Jinrong Peng (Zhejiang University, Hangzhou) reported the function of the nucleolar protein Def in zebrafish liver regeneration, noting that some newly formed hepatocytes are derived from non-hepatocytes by Cre-loxP based lineage tracing. Jie He (Institute of Neuroscience, Shanghai) presented the trans-differentiation of tectal radial glia cell fates into glial cells in response to stab injury at different life stages in zebrafish (Yu and He 2019). Ping Hu (Bioland Laboratory, Guangzhou) investigated the potential differentiation of muscle stem cells into tendons in vitro. Feng Liu (Institute of Zoology, Beijing) shared his recent studies on fate determination and expansion of hematopoietic stem cells in zebrafish and mice (Xia et al. 2021). Anming Meng (Tsinghua University, Beijing) presented an inspiring work about the origin of hematopoietic stem cells from the somites in zebrafish and mice. Ye-Guang Chen (Tsinghua University, Beijing) showed that the loss of bone morphogenetic protein signaling results in the expansion of intestinal stem cells due to activation of the AKT pathway, and the critical role of different cell types and signaling pathways in stem cell renewal and gastrointestinal homeostasis. And Dahai Zhu (Bioland Laboratory, Guangzhou) presented fascinating work related to the metabolism-mediated translational control of muscle stem cell activation and regeneration.

In addition to stem-cell based regeneration, the induction of quiescent cells into proliferation provides another cell source for organ regeneration. Yiting Zhou (Zhejiang University, Hangzhou) showed that one type of cell fate determination is achieved by non-epigenetic-dependent metabolic remodeling. Ke Wei (Tongji University, Shanghai) presented his work on high-content chemical screening for small molecules that induce iPSC-derived cardiomyocyte proliferation, noting that the L-type Ca^2+^ channel blocker, nimodipine, is able to induce DNA synthesis. Weida Li (Tongji University, Shanghai) applied single-cell RNA sequencing to tease out the molecular mechanisms of the maturation of human embryonic stem cell-derived beta-cells, leading to the intriguing finding that ZnT8 blocks the release of Zn ions to prevent beta-cell maturation. In addition, Suhong Xu (Zhejiang University, Hangzhou) reported an interesting work on cell plasma membrane repair after laser ablation in *Caenorhabditis elegans*. And Guangshuo Ou (Tsinghua University, Beijing) revealed that sphingolipid is required for cilia regeneration.

### Injury-induced inflammatory signals

The immune cells, as the early responsive cell types, play an important role in the clearance of dead cells and the regeneration of new ones, engaging at a number of processes including damage-associated cell clearance, inflammation, revascularization, and fibrotic scar formation/resolution. The outcome of inflammatory responses can be complex and bidirectional between restoring tissue integrity and chronic tissue damage. Ting Chen (National Institute of Biological Sciences, Beijing) applied single-cell RNA sequencing to dissect the pathological mechanisms underlying vitiligo, noting that the interferon signaling in fibroblasts is crucial for the recruitment of CD8-positive immune cells that attack melanocytes. And Jing-Wei Xiong (Peking University, Beijing) proposed that Dusp6-MAPK signaling regulates heart regeneration in both zebrafish and mammals, noting that the Dusp6-MAPK pathway fine-tunes neutrophil-mediated cardiac damage while macrophage Dusp6 regulates cardiac fibrosis in post-myocardial infarction in rats.

### Other regenerative signaling pathways

While considerable progress has been made in regenerative processes, it remains largely unknown what the underlying molecular mechanisms are among different cellular components during organ regeneration. It is believed that intercellular signaling is essential for achieving seamless organ repair and regeneration. Ying Xi (Shanghai University of Science and Technology, Shanghai) presented a fascinating work on the role of hypoxia in alveolar regeneration (Xi et al. 2017). Hai Huang (Zhejiang University, Hangzhou) presented his recent work on *Drosophila* tracheal remodeling, noting that both YKI and ERK signaling regulate the migration and proliferation of tracheal progenitors. Dong Liu (Southern University of Science and Technology, Shenzhen) reported the critical role of Wnt-Sim1b signaling in zebrafish hair cell regeneration. Lingfei Luo (Southwest University, Chongqing) presented his recent work on the role of lymphatic vessels in zebrafish cerebral vessel regeneration, noting that lymphatic vessels pioneer to pave the way for or directly transdifferentiate into newly-formed vascular endothelial cells after injury (Chen et al. 2019). Hai Song (Zhejiang University, Hangzhou) presented the role of the Hippo pathway in CCM formation, demonstrating how MEKK3 kinase regulates the interaction of YAP and KLF4 to promote CCM pathogenesis (Lu et al. 2021). Tao Zhong (East China Normal University, Shanghai) presented the role of Grl/Hey2, an important angiogenic signaling, in zebrafish heart regeneration (She et al. 2020). Jie Wang (Guangzhou Institutes of Biomedicine and Health) presented his work on retinal regeneration from the Muller glia. Yungui Yang (Institute of Genomics, Beijing) presented his work on m6A function in planarian regeneration, noting that m6A modifications of RNA are enriched in the regenerating cell clusters. Wei Wang (National Institute of Biological Sciences, Beijing) discussed about his recent work on the identification and function of regenerative enhancers in killifish and zebrafish (Wang et al. 2020). Zhicao Yue (Shenzhen University, Shenzhen) presented his work on how hair-follicle repair and regeneration is affected by environmental factors, noting that the chilling pre-condition promotes while the warming pre-condition blocks repair. And Heng Wang (Huazhong Agriculture University, Wuhan) showed that blastema formation during salamander limb regeneration is influenced by extreme starvation.

### Advanced technologies applied to organ regeneration

The development of biological sciences, including regenerative biology, has dramatically benefited from new technologies. During the past decade, we all witnessed the emergence of single-cell genomic technology and how it revolutionized our studies of cellular mechanisms from a variety of biological samples including humans. Shanshan Ai (Southern Medical University, Guangzhou) presented the application of single-cell itChIP-seq and CoBATCH for the study of genomic regulation and epigenetic dynamics during endothelial cell lineage specification (Ai et al. 2019). Bin Zhou (Center for Excellence in Molecular Cell Sciences, Shanghai) presented a series of lineage-tracing systems with the combination of two site-specific recombinases (Cre and Dre) and found that newly-formed beta cells are mainly derived from the transdifferentiation of pre-existing non-beta cells but not progenitor cells (Zhao et al. 2021). Gong Chen (Jinan University, Guangzhou) presented their work on promoting the transdifferentiation of endogenous astrocytes into functional neurons by over-expression of NeuroD1, which are promising for gene therapy in a rhesus monkey stroke model (Chen et al. 2020). Small molecules feature multiple targets, easy delivery, scalable doses and timing, and low immunogenicity, therefore they are popularly applied to the study of biological processes including cellular reprogramming. Hongkui Deng (Peking University, Beijing) presented their exciting work on the in situ transdifferentiation of astrocytes into neurons by small molecules (Ma et al. 2021). And Yang Zhao (Peking University, Beijing) presented a chemical screen for small molecules enabling fibroblast transdifferentiation into hepatocytes.

In the past two decades, significant progress has been made in creating prototypes of tissue substitutes such as nerve conduits, blood vessels, livers, and even hearts (Berthiaume et al. 2011). Jian Luo (East China Normal University, Shanghai) presented his work on cartilage regeneration, noting that disruption of EP4 promotes articular cartilage regeneration by enhancing chondrogenesis. Qi Gu (Institute of Zoology, Beijing) presented his recent work on bioinspired engineering by applying collagen grafts to polydimethylsiloxane to establish a uterus-inspired 3D niche. And Rongrong Zhu (Tongji University, Shanghai) showed her findings that layered double hydroxide (LDH), a type of biomaterials, helps embryonic stem cell self-renewal/pluripotency and spinal cord repair (He et al. 2021b; Zhu et al. 2021).

In summary, this meeting brought together many biologists, physicians, and engineers to discuss the mechanisms underlying organ regeneration and potential applications of animal regeneration studies to human diseases. We were excited to witness that more young principal investigators joining the field of regenerative biology in China. The exploration of regeneration requires more talented investigators and multiple-disciplinary efforts to move the field forward. While this meeting covered quite extensive spectrum of animal species, it will be important to have more investigators from the fields of plant biology and human organoids to attend future meetings. We anticipate that this series of organ regeneration conferences will also become a good channel for interaction with international colleagues in this exciting field over the coming decade.

## Data Availability

Not applicable.
